# Feasibility of a multidimensional interdisciplinary rehabilitation program for people with dementia and their informal primary caregivers – a randomized pilot trial with a caregiver focus

**DOI:** 10.1186/s12877-026-08532-1

**Published:** 2026-10-21

**Authors:** Mia Conradsson, Birgitta Olofsson, Henrik Holmberg, Nina Lindelöf, Ingeborg Nilsson, Yngve Gustafson, Håkan Littbrand

**Affiliations:** 1 Department of Community Medicine and Rehabilitation, Umeå University, Umeå, Sweden; 2 Department of Nursing, Umeå University, Umeå, Sweden; 3 Department of Diagnostics and Intervention, Orthopedics, Umeå University, Umeå, Sweden; 4 Department of Epidemiology and Global Health, Umeå University, Umeå, Sweden

**Keywords:** Caregiver burden, Cognition disorders, Depression, Intervention study, Quality of life

## Abstract

**Background:**

To improve the situation of informal caregivers, both supportive efforts for caregiver and rehabilitation for the person with dementia, may be needed. This randomized controlled pilot trial evaluated, among caregivers, the feasibility of a person-centred multidimensional interdisciplinary rehabilitation program for community-dwelling people with dementia that incorporated an education and support intervention for informal caregivers.

**Methods:**

Informal caregivers were randomized alongside the person with dementia to an intervention group (n=36) or usual care (n=31). Multiprofessional team rehabilitation for individuals with dementia and support for caregivers, were offered for one 20-week and two 4-week follow-up periods after 5 and 14 months. Caregivers were offered assessments, participation in group sessions, and individual support. After the last group session, scheduled during the last four weeks of the 20-week period, a survey was distributed. Attendance at group sessions and uptake of individual support were recorded. Blinded assessments of caregiver burden (Caregiver Burden Scale, CBS), depressive symptoms (Geriatric Depression Scale 15-item version, GDS) and health-related quality of life (Short Form 36–Health Survey, SF-36), were performed at baseline and after 5, 12, 24, and 36 months.

**Results:**

Thirty-seven participants (56%) were spouses and 26 (39%) were adult children. Participation rate in assessments of outcomes at 36 months was 100% and 87% for intervention and control group in GDS and SF-36, respectively, and 69% and 61% for CBS, respectively. Missing data were primarily due to mortality. All caregivers in the intervention group (n=36) were assessed, and all received individual interventions by team staff. Twenty-three caregivers attended group sessions, and 21 completed a survey after the last session. Twenty (95%) caregivers reported increased knowledge about supporting the person with dementia, and 19 (90%) improved coping strategies. No significant differences were observed between groups in depressive symptoms or caregiver burden, yet differences in favour of the control group were seen in subscales of health-related quality of life at 24 and 36 months.

**Conclusions:**

The program, combining rehabilitation for the person with dementia with caregiver support, seems feasible regarding caregivers’ participation in the intervention and study evaluation, and thus further evaluation in an adequately powered randomized controlled trial seems relevant. A future definitive trial should evaluate continuous support over a longer period adapted to the caregiver and refine assessment procedures, including conducting assessments during ongoing interventions to provide greater insights into rehabilitation and caregiver-support effects.

**Trial registration:**

The study protocol is published online 04/11/2015 at http://www.isrctn.com/ISRCTN59155421.

**Supplementary Information:**

The online version contains supplementary material available at https://doi.org/10.1186/s12877-026-08532-1.

## Background

Dementia has a major impact on the affected person but also on the people in their close network. The majority of individuals with dementia live in the community [1, 2] so in addition to experiencing the effects of dementia on a close relative, family and friends often take on the role of informal caregivers. While caregiving can be rewarding and meaningful, it can also be demanding. Caring for a person with dementia seems to be the most demanding type of caregiving [3, 4], and may burden caregivers [5] and lead to negative consequences on their mental and physical health, quality of life, finances, participation in society and survival [6, 7]. Caregivers provide most of the assistance in fulfilling the basic needs of the person with dementia, addressing both instrumental and personal activities of daily living (ADL), as well as their participation in society, and the extent of this support increases gradually because of the progressive course of the disorder. The WHO Global Dementia Action Plan 2017–2025 highlights the importance of providing support and training to improve informal caregivers’ knowledge and caregiving skills, to prevent stress and health problems, and also to enable persons with dementia to live in the community [8].

Caregiver burden is influenced by caregiver characteristics including age, gender, education, and personal coping style, as well as by characteristics of the person with dementia. Particularly neuropsychiatric symptoms and functional disabilities are important determinants of caregiver burden, depression, and mental health status [9–12]. Thus, support is needed for caregivers. However, to influence the caregiving situation, it seems necessary to also target interventions to the person with dementia. If the condition can be optimized, the lives of both the person with dementia and the caregiver might be improved. The many and complex consequences of dementia for the affected individual imply the need for person-centred interdisciplinary rehabilitation efforts. Subsequently, recent recommendations emphasize multiprofessional rehabilitation programs as a fundamental service when it comes to meeting the needs of people with dementia [8, 13, 14]. However, to date, multiprofessional rehabilitation in this population is not routinely available in clinical settings [15–17], and scientific knowledge is limited [18, 19].

Intervention studies aimed at caregivers are numerous and several systematic reviews have synthesized their effects [20–22]. Supportive interventions seem to positively influence caregiver burden, depressive symptoms, and well-being among informal caregivers; however, the effect sizes are small to moderate [22]. The most effective interventions seem to be multicomponent interventions involving for example a combination of education, support, problem solving, and skills training, as well as psychoeducational interventions involving active participation [20–22]. Several studies have evaluated dyadic efforts, i.e. efforts aimed at both the caregiver and the person with dementia, but these have not indicated additional effects compared to caregiver support alone [20]. The existing dyadic studies predominantly evaluate single interventions aimed solely at the individual with dementia, or joint support initiatives directed simultaneously at both the person with dementia and their caregiver. Thus, the impact of multiprofessional rehabilitative interventions specifically tailored to the person with dementia combined with targeted support for caregivers remains insufficiently evaluated regarding outcomes for the caregiver.

The Multidimensional Interdisciplinary Rehabilitation in Dementia (MIDRED) is a randomized controlled pilot study evaluating the feasibility of a person-centred rehabilitation program for community-dwelling older people with dementia, which incorporated an education and support intervention for informal primary caregivers. As earlier published, evaluation through qualitative interviews with the individuals with dementia in the MIDRED study conveyed that they found the program feasible and advantageous, and experienced noticeable improvements [23]. Moreover, following interviews with the team staff, it was determined that within a comprehensive multiprofessional team, it was possible to deliver personalized rehabilitation through creative collaboration with the individuals affected by dementia and their informal caregivers [24]. In addition, the program was feasible among participants with dementia and showed potential clinical meaningful findings in the areas of social participation, short-term depressive symptoms, gait, physical activity and neuropsychiatric symptoms, as well as their ability to continue living in the community [25, 26]. In qualitative interviews with informal caregivers in the MIDRED study, they described participation in the program as valuable – both for themselves and their relative with dementia [27]. The aim of the present study was to further evaluate the feasibility of the MIDRED rehabilitation program among informal caregivers using quantitative methods; i.e., to explore if a definitive randomized controlled trial (RCT) can be done, should be done and, if so, how. Specifically, feasibility was evaluated in terms of follow-up rates, adherence and delivery of assessments and interventions, a survey evaluation of group sessions, and potential effects. Potential short- and long-term effects on caregiver burden, depressive symptoms and quality of life among informal primary caregivers were evaluated, and estimates of data required to design a definite randomized controlled trial reported.

## Methods

### Setting and participants

The study was part of the MIDRED study, a randomized controlled pilot trial with parallel group design, undertaken in Umeå, Sweden from 2016 to 2019. The present study focused on quantitative data concerning the informal primary caregivers in the MIDRED study.

Community-dwelling people with a dementia diagnosis, and their informal caregivers, were located through the County Council of Västerbotten and recruited, from November 4th, 2015, to January 21st, 2016, via local health centres in Umeå or the outpatient unit of the Geriatric Center, at the University Hospital in Umeå [25]. Staff at the six different participating units contacted potential participants with dementia. Upon approval, study staff disseminated study information, contacted potential participants by telephone, and performed a home visit to assess whether the person met the inclusion criteria, see Hasselgren et al. for details [25]. A maximum of two informal caregivers (people within the family or relatives, or a friend or neighbour involved in the care or support of the person with dementia), were invited to participate. Informal caregivers are hereby referred to as ‘participants’ in the present study. 

### Sample size and randomization

Power calculations based on the primary outcome of the MIDRED study (proportion of people with dementia continuing to live in the community at the 24-month follow-up assessment), estimated a total sample of 179 persons with dementia for the study [25]. However, due to limited funding only the first of three pre-planned waves were completed, (61 persons with dementia together with 67 informal caregivers), and the study is therefore reported as a pilot trial [28]. The decision to discontinue further recruitment waves was made in February 2019, once the 36-month follow-up data for the first wave had been fully collected. Informal caregivers participated alongside the persons with dementia, who were randomized with a 1:1 allocation to a control group (usual care) or a person-centred multidimensional interdisciplinary rehabilitation program. Randomization was stratified (type of dementia and household living conditions) to obtain an equal distribution of factors related to the person with dementia which was considered important in view of the study’s primary outcome, see Hasselgren et al. for details [25].To eliminate the possibilities of selection bias (ensuring allocation concealment), two PhD-students not involved in the study performed the randomization after the inclusion process and baseline assessments were finished. Using a die, participants were randomized in blocks of two within each stratum to maintain balance between intervention/control.

### Rehabilitation program

Informal caregivers and persons with dementia in the intervention group were offered a 20-week rehabilitation period (February-June 2016), and two additional follow-up periods of four weeks each, five and 14 months after the 20-week period. Rehabilitation consisted of assessments and interventions by a comprehensive multiprofessional team. The team included two physiotherapists, two occupational therapists, one social worker, and one assistant nurse working full time; and two physicians, one nurse, one dietician, one neuropsychologist, one dental hygienist, and one clinical pharmacist working part-time. The team staff were all experienced in working with older people with dementia and physical impairments and in practicing person-centred care [29]. For more information about the intervention program offered to individuals with dementia, see Hasselgren et al. [25].

#### Rehabilitation program aimed at informal caregivers

The program comprised multicomponent interventions including education, support, active participation, and problem solving [20–22, 30]. The intervention was guided by a self-management framework aimed at enhancing caregivers’ knowledge and self-management skills [31]. As a first step during a four-week assessment period, the social worker assessed the need for support and counselling through interviews with participants either during a home visit or by telephone. The assessment covered family and living circumstances; the person with dementia´s level of disability and care needs; community services received; caregiving duration; caregiver health; and support from family or friends. It also addressed participants´ support needs, potential benefits for the person with dementia, and plans for ongoing support from the social worker during the rehabilitation period.

##### Individual support to informal caregivers

Following the initial assessments, the social worker offered up to six individual support and counselling sessions over 16 weeks. Support was tailored to individual needs and included information on formal care services, financial and psychological support, and strategies for managing behavioural symptoms. Other team professionals provided additional interventions as needed to support the participants in their caregiving role.

##### Group sessions for informal caregivers

The social worker offered face-to-face group sessions at the Geriatric Centre, Umeå University Hospital. Groups were formed based on relationships to the person with dementia (partner/sibling, adult child). To facilitate attendance among working caregivers, sessions for adult children were scheduled in the late afternoon. Sessions lasted approximately 90 min, once a week for six weeks, combining education and group discussions [30]. Guided by a self-management framework, sessions aimed to enhance caregivers’ knowledge and self-management skills [31]. Topics were tailored to participants’ needs and included the characteristics and the course of dementia disorder; the available community services; how to prevent and deal with behavioural symptoms; and recommendations for health-promoting activities, including physical activity, nutrition, oral health, and participation in society (Table A1, Additional file 1). Sessions also aimed to facilitate social networking among participants. The participants and group leaders jointly explored strategies to address caregiving challenges. Sessions were led by the social worker and/or the nurse. In four sessions, professionals in the rehabilitation team (physician; neuropsychologist; physiotherapist and occupational therapist; and dietician and dental hygienist), provided oral and written information, and joined in the group discussion.

#### Rehabilitation program aimed at persons with dementia

During the initial assessments of the person with dementia, team staff identified problems, needs, and strengths in 10 areas: functional capacity, cognitive function, ADL performance, falls, participation in society, physical activity, nutrition, medical conditions, neuropsychiatric symptoms (NPS), and drug use. The findings were used to determine each person with dementia´s intervention needs from each professional in the team. In collaboration with each person with dementia, his/her informal caregiver(s), and representatives of team staff, individual rehabilitation goals and follow-up plans were established. Based on these goals, relevant professionals formed an individualized rehabilitation team and developed a tailored intervention plan.

The persons with dementia were offered individualized interventions based on the identified problems and the rehabilitation goals, delivered by relevant profession in the team. Interventions were conducted at the Geriatric Centre day rehabilitation unit, in the person with dementias´ home, and/or in community settings. Individual interventions included e.g. cognitive technical devices; support to engage in household activities; guidance for achieving recommended physical activity levels; medication reviews; nutritional and oral health support; medical follow-up; as well as introducing the person with dementia to a day-care centre and other activities in the community. In addition, the person with dementia was offered twice weekly individual exercise for 16 weeks, at the geriatric day rehabilitation unit, followed by a social coffee break. Sessions were conducted in small groups, supervised by physiotherapists, and based on the High-Intensity Functional Exercise program [32]. The assistant nurse organized transportation and assisted the person with dementia during the visit to the Geriatric Centre.

#### Follow-up by team staff after the 20-week rehabilitation period

Five and 14 months after the rehabilitation period of 20 weeks, the participants and the person with dementia were followed up by the team staff. For a duration of four weeks, the team staff made assessments in the same manner as at the start of the study, earlier recommended interventions were followed up on, and complementary interventions could be initiated if needed.

### Control group

Participants in the control group – both caregivers and persons with dementia – continued their usual care i.e., they continued their ordinary contacts with the healthcare system according to their needs and planned follow-ups by primary care, as well as with efforts from the community and social services.

### Measurements

The MIDRED study´s outcome measures were assessed at five occasions: at baseline (January 2016) and at 5 (after the intervention), 12, 24 and 36 months after baseline, respectively. The assessor was blinded to group allocation, and a specific strategy to preserve blinding throughout the study was developed in advance [25]. If blinding was broken anyway, the assessor was replaced according to a schedule, to enable all tests to be performed blind. A booklet containing questionnaires for the participants was distributed either by the person interviewing and assessing the person with dementia when the participants were present if possible, or it was sent by post or delivered to a postal box by project staff. Instructions for each specific questionnaire were provided within the booklet. Descriptive data were collected regarding age, sex, living condition, and the relationship to the person with dementia. A specialist in geriatric medicine (YG) reviewed all test protocols at the follow-ups, to assess whether any participant (intervention or control) had indications of serious health issues, e.g. symptoms of depression. In those cases, the participants were contacted specifically by telephone and offered information about test results and recommendations to contact health care.

#### Adherence and delivery of assessments and interventions to participants

Individual assessments and interventions made by the team staff directed at the participants and the individuals with dementia were documented in medical records of the person with dementia during the 20-week period and the two follow-ups for the intervention group. For people with dementia in both the intervention and the control group, data on all medical, dental, and rehabilitative assessments and interventions, including support given to caregivers, performed in any outpatient setting during the 20-week rehabilitation period and the two follow-up periods, were collected from medical and dental care records [33]. Type of assessment or intervention was coded, and more than one type could be carried out during one session. Attendance at group sessions was recorded.

#### Survey evaluation of group sessions

After the last group session, scheduled during the last four weeks of the 20-week period, a survey was distributed to participants who had joined the group sessions. The survey was filled out anonymously. Seven questions concerned the format of sessions (including sessions number, length, content, and spacing) and two concerned impact on participants´ life situation. The two questions concerning impact of sessions were: *Has the information and discussions in the family caregiver support group resulted in any changes in your ability*: (1) *to cope with your life situation?* (2) *to provide support to your relative with dementia?* (answers: yes, positive impact; yes, negative impact; no impact; I don’t know).

#### Outcome measures of potential effects

Potential effects were evaluated in the areas of caregiver burden, depressive symptoms, and health-related quality of life. Caregiver burden was assessed using the Caregiver Burden Scale (CBS) [34], measuring subjective experienced burden of caring for a person with a chronic disease. The scale has 22 questions answered by marking one of four response categories ranging from 1 to 4 (not at all, seldom, sometimes, or often). The scale is also divided into five subscales: general strain, isolation, disappointment, emotional involvement, and environment. A mean value is calculated for the total score, as well as for subscales ranging from 1 to 4 points, and the severity of burden can be interpreted as 1.00–1.99 (low burden), 2.00–2.99 (moderate burden) and 3.00–3.99 (high burden) [3]. The Swedish version has been shown to be valid and reliable among informal caregivers of people with dementia [34].

Depressive symptoms were assessed using the 15-item Swedish version of the Geriatric Depression Scale [35–37]. The scale has fifteen questions in a yes/no format. The score ranges from 0 to 15 and a score of 0–4 is considered as falling within the normal range, 5–9 indicates mild depression, and a score of 10 or more indicates moderate to severe depression [38]. Participants were asked to rate their status during the last two weeks.

The Short Form 36–Health Survey (SF-36) measured health-related quality of life. The scale includes aspects of physical and psychological health and social well-being among adults, according to WHO´s definition of health. It comprises 36 questions in a Likert scale format. The responses to 35 questions are interpreted over eight dimensions all strongly correlated to disease: physical function, role limitations due to physical health problems, bodily pain, general health perceptions, vitality, social functioning, role limitations due to emotional problems, and general mental health. The question in SF-36 assessing health change during the last year was not used in the present study. The Swedish version of the SF-36 v2 was used, which has shown essentially the same psychometric properties as earlier versions [39]. The questions covered the participants´ state over the last four weeks. Scores in subscales are weighted into a sum ranging from 0 to 100.

#### Baseline and descriptive measurements of the person with dementia

The person with dementia was interviewed and assessed in their home. Information on diagnoses and prescribed drugs was collected by reviewing medical records. All diagnoses were verified by a physician specialized in geriatric medicine (YG). The physician used electronic records, current pharmaceutical treatment, and assessment results to confirm depression and dementia diagnoses according to the Diagnostic and Statistical Manual of Mental Disorders, 4th edition, text revision [40]. Cognitive function was assessed using the Mini-Mental State Examination (0–30 points) [41]. The assessor interviewed an informal caregiver or care staff who knew the person well, regarding the ability to manage ADL and the presence of neuropsychiatric symptoms. Ability to manage personal and instrumental ADL (p- and i-ADL) was assessed using the Lawton & Brody scale [42, 43] and the Functional Independence Measure [43]. The presence of neuropsychiatric symptoms was assessed using Neuropsychiatric Inventory (NPI, range 0–144 points, higher scores indicate more symptoms) [44]. The NPI items were divided and presented in the following subsyndromes; *hyperactivity* (agitation, disinhibition, irritability, euphoria, aberrant motor behaviour, 0–60 points), *affective* (depression, anxiety, 0–24 points), *apathy* (apathy, appetite, 0–24), *psychosis* (delusions, hallucinations, night-time behaviour 0–36 points) [45]. Use of social services was retrieved from records from the municipality according to the Social Services Act.

### Statistical analysis

All analyses were performed according to the intention-to-treat principle, using all available data according to original group allocation, regardless of the level of attendance in the intervention. Data from survey questions are presented as proportions of participants in the intervention group. The SF-36 questions were entered into a statistical software distributed by Quality Metrics Inc, and sum scores for the eight original health dimensions were retrieved and further entered in the SPSS version 24 (IBM Corporation, Armonk, NY). Due to criticism towards the algorithm for the global component scores of the SF-36, scores for physical and mental health were calculated as the mean of the raw scores from the original dimensions in the questionnaire [46]. The physical component score (PCS raw) was based on physical functioning, physical role functioning, bodily pain, and general health perceptions; and the mental component score (MCS raw) was based on vitality, social functioning, emotional role functioning, and general mental health.

Missing values were imputed into GDS and CBS, including the subscales, when at least 2/3 of the questions were answered. The missing value was imputed with the mean of the answered questions, and the total score was recalculated and rounded up to an integer [47]. For GDS, imputations were made for a total of 22/320 questionnaires imputing 1–3 questions. Corresponding figures for CBS were 4/289 questionnaires with one missing value each. For the dimensions in SF-36, imputations were made in 11/321 questionnaires with 1–2 missing values, according to the PRO Core software using Missing Score Estimation which incorporates algorithms for calculating scores for partially completed surveys.

Imbalances between the groups (intervention and control) in descriptive characteristics in Table 1 were evaluated using an independent samples t-test for continuous variables and chi-square test for categorical variables. Longitudinal changes in the outcome measures were analysed using linear mixed-effect models with interaction terms for group and time-point. The analysis considers the baseline value as the first time-point in the outcome variable in order to use all available data. The analyses were adjusted for age and sex as fixed effects and individual as random effects. All analyses were performed using IBM SPSS Statistics, version 28. All statistical tests were two-tailed, and *p* < 0.05 was considered statistically significant.

## Results

### Participants

**Fig. 1:**
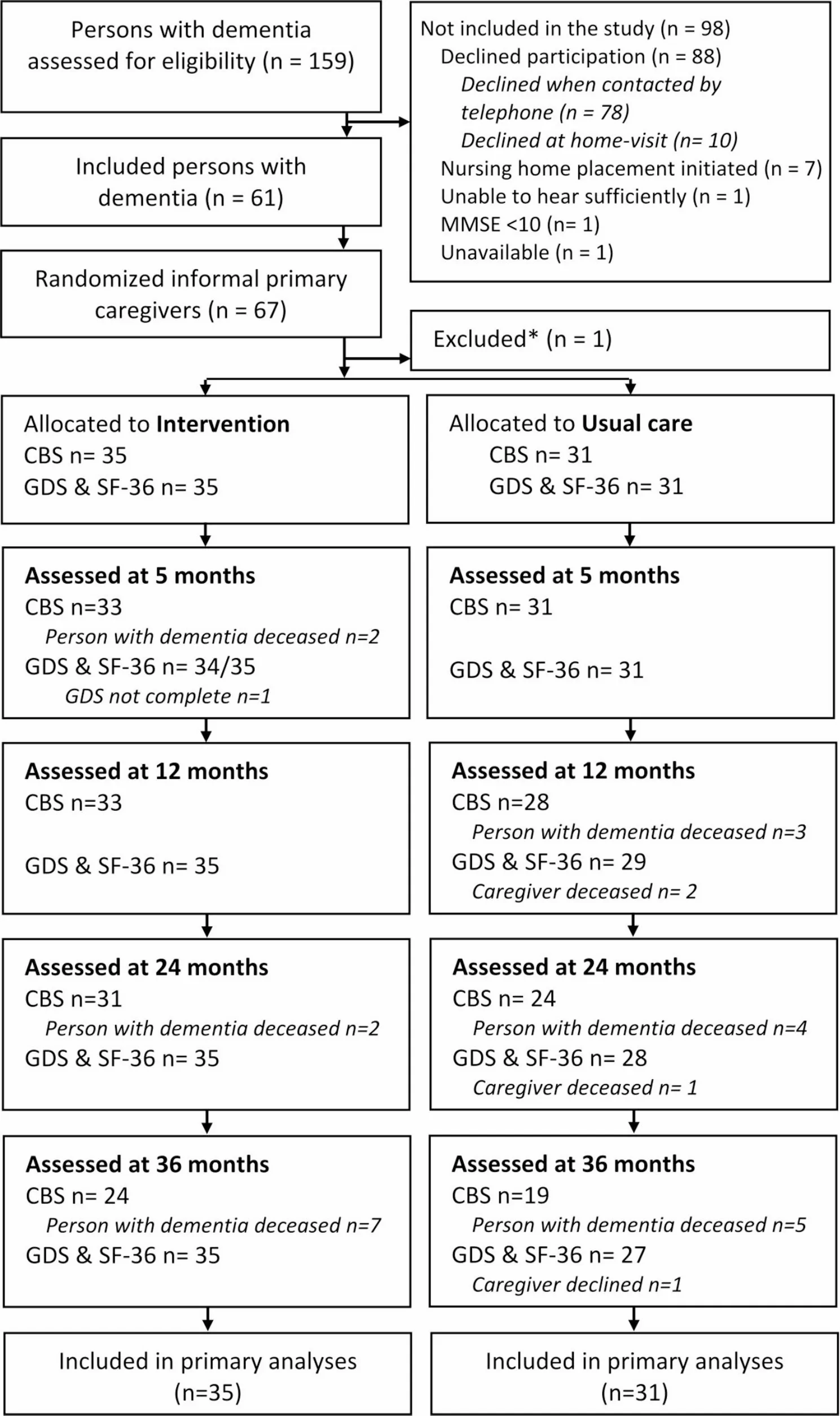
Flow chart of the persons with dementia and the informal caregivers through the study. CBS = Caregiver Burden Scale, GDS = Geriatric depression scale, SF-36 = The Short Form 36 –Health Survey. *Excluded due to removal of dementia diagnosis from person with dementia at the 36-month follow-up

The inclusion of participants is displayed in the flowchart in Fig. 1. After the study was completed at 36 months, one person with dementia and the informal caregiver were excluded from the study since it emerged from medical records that the Alzheimer disease diagnosis had been removed. Thus, analyses in the study are comprised of 66 informal caregivers. Baseline characteristics of the participants included are presented in Table 1, and for persons with dementia in Table A2, Additional file 2. Informal caregivers had a mean age (range) of 66.9 (30–90) years, 39 (58%) were women and 39 (59%) were living together with the person with dementia. Thirty-seven participants (56%) were spouses, 26 (39%) were adult children, two (3%) were siblings and one (1.5%) was a niece/nephew. Sixteen (24%) had GDS scores ≥ 5, indicating symptoms of depression, and 36 (55%) had CBS total scores ≥ 2.0, indicating moderate or high caregiver burden. The intervention group had higher baseline scores, indicating better health, on three dimensions of the SF-36: physical function, energy/fatigue or vitality, and general mental health, as well as on the summarized physical and mental component raw scores.

**Table 1:** Baseline characteristics of informal primary caregivers in the MIDRED study (*n* = 66)

Characteristic	Total *n* = 66	Control group (*n* = 31)	Intervention group (*n* = 35)	*p*-value
Age, mean ± SD /median (range)	66.9 ± 14.0/68.5 (30–90)	67.1 ± 14.7	66.7 ± 13.0	0.90
Women, n (%)	39 (59)	20 (65)	19 (54)	0.40
Living together, n (%)	39 (59)	18 (58)	21 (60)	0.87
Spouse/Partner, n (%)	37 (56)	17 (55)	20 (57)	0.85
**Outcome measures**, mean ± SD (range)				
Geriatric Depression Scale (0–15)	2.78 ± 3.05 (0–13)	3.19 ± 2.8	2.20 ± 2.3	0.12
Geriatric Depression Scale > 4, n (%)	16 (24)	9 (29)	7 (20)	0.39
Caregiver Burden Scale (CBS), (1–4)	2.03 ± 0.47 (1–3)	2.08 ± 0.41	2.00 ± 0.52	0.47
Moderate or high burden CBS > 2.0, n (%)	36 (55)	19 (61)	17 (49)	0.30
- *General strain*	2.24 ± 0.56 (1–3.4)	2.29 ± 0.48	2.20 ± 0.62	0.51
- *Isolation*	2.0 ± 0.69 (1–4)	2.01 ± 0.68	1.99 ± 0.71	0.91
- *Disappointment*	1.83 ± 0.55 (1–3.2)	1.83 ± 0.56	1.83 ± 0.54	0.95
- *Emotional involvement*	2.11 ± 0.67 (1–3.3)	2.22 ± 0.56	2.02 ± 0.75	0.23
- *Environment*	1.81 ± 0.62 (1–3.7)	1.90 ± 0.60	1.73 ± 0.64	0.27
SF-36 (0–100)				
Physical Component Score, raw sum (PCS)	75.1 ± 21.0 (14–100)	69.1 ± 25 (14–100)	80.4 ± 16 (45–100)	0.03
- *Physical Function (PF)*	82.2 ± 21.5 (10–100)	75.2 ± 26.4	88.4 ± 13.7	0.02
- *Role limitations due to physical health problems (RP)*	81.5 ± 24 (0–100)	75.6 ± 29.3	86.8 ± 17.9	0.07
- *Bodily pain (BP)*	68.7 ± 26 (12–100)	62.7 ± 28.5	73.9 ± 23.4	0.08
- *General health perceptions (GH)*	68.1 ± 23 (5–100)	63.1 ± 26.5	72.6 ± 19.5	0.10
Mental Component Score raw sum (MCS)	74.5 ± 18 (24–100)	69.8 ± 21 (24–100)	78.7 ± 13 (39–98)	0.047
- *Energy/fatigue or vitality (VIT)*	59.8 ± 23 (0–100)	53.7 ± 25.6	65.2 ± 19.6	0.048
- *Social functioning (SF)*	82.0 ± 18 (25–100)	78.2 ± 20.9	85.3 ± 15.3	0.12
- *Role limitations due to personal or emotional problems (RE)*	79.8 ± 26 (0–100)	75.0 ± 31.8	84.0 ± 18.8	0.17
- *General mental health (MH)*	76.4 ± 15 (30–100)	72.2 ± 17.4	80.1 ± 13.1	0.044

Note: For the Geriatric Depression Scale and Caregiver Burden Scale, higher scores indicate more depressive symptoms and more burden, respectively. For Short Form 36–Health Survey (SF-36), higher scores indicate better health. Differences between activity groups were analysed using the χ2 or independent samples t-test. SD = Standard Deviation

At study start, the 60 persons with dementia – 35 women and 25 men – had a mean ± standard deviation (SD) age of 78.9 ± 6.4, and a mean ± SD MMSE score of 20.9 ± 3.9 (Table A2, Additional file 2). Twenty-nine (48%) had Alzheimer’s dementia, 11 (18%) had vascular dementia, 11 (18%) had mixed Alzheimer’s and vascular dementia, and nine (15%) had other types of dementia. According to the Lawton ADL-index, 16 (27%) were independent in p-ADL. Mean scores of NPI were 9.7 and ranged from 0 to 48 points. Twenty (33%) had home-help services, and nine participants (15%) went regularly to day-centre activities. Table A3, Additional file 3, shows the use of social services, including home-help services over 36 months, divided into control and intervention groups.

### Adherence to measurements at follow-ups

Proportions of participants filling out the three questionnaires at 5, 12, 24, and 36 months ranged 64–66/66 (97–100%), 61–64/66 (92–96%), 55–63/66 (83–95%), and 43–62/66 (65–94%), respectively. Reasons for missing assessments are displayed in Fig. 1. For caregivers in the intervention group, the median (interquartile range [IQR]) number of days between the ending of the 20-week period and filling out questionnaires at the 5-month follow-up, was 12 (6–13). Because one participant had severe visual impairment, the questionnaires were read out loud by the same assessor testing the person with dementia.

### Adherence and delivery of assessments and interventions to participants

Of the participants in the intervention group, 35/35 (100%) received assessments by the social worker, and 35/35 (100%) received interventions during the 20-week rehabilitation period. Of the 131 interventions given during the 20-week rehabilitation period, 121/131 (92%) were given by the social worker, and 10 (8%) by an occupational therapist, dietician, physician, neuropsychologist, nurse, or dental hygienist. The number of interventions aimed at partners was 87/131 (66%), adult children 36/131 (27%), and siblings/niece/nephew 9/131 (7%). For the first follow-up, 33/33 (100%) were assessed by the social worker, 32/33 (97%) received interventions, and 46/47 (98%) of interventions were delivered by the social worker. Corresponding figures for the second follow-up were 32/32 (100%) receiving assessments, 32/32 (100%) receiving interventions, and 37/37 (100%) of interventions were delivered by the social worker. The median (IQR) for number of interventions among those caregivers receiving interventions were 3 (2–5) for the 20-week period, 1 (1–2) for the first follow-up, and 1 (1–1) for the second follow-up.

Of the 35 caregivers in the intervention group, 23 (66%) participated in group sessions. Participation rate among partners and siblings were 15/21, and 8/13 among adult children. They were divided into one group with adult children and two groups with partners and siblings of the person with dementia, and number of participants per group ranged from 7 to 8. The overall attendance in the group sessions was in mean 87% (120/138 sessions), and 19/23 (83%) of the participants attended five or more out of six occasions. One participant was not invited to the group sessions due to a misunderstanding in the communication between the research group and team staff at the study start. Instead, the participant had a private session with the social worker to take part in the material, including written hand-outs from their sessions of choice, and was offered individual support by the social worker according to the intervention plan.

### Survey evaluation of group sessions

Most participants considered the number of sessions (81%), interval between sessions (95%), and session duration (100%), to be adequate. The content was rated as very good by 90% of participants, and 90% also reported that no topic had been covered either excessively or insufficiently. Regarding content, 43% indicated that additional topics could have been included, and 29% reported that no topics were missing. Twenty (95%) caregivers reported a positive impact on their ability to support the person with dementia, and one registered “I don’t know”. Further, 19 (90%) caregivers noted a “positive impact” on their ability to cope with their life situation, one reported “no impact” and one registered “I don’t know”.

### Outcomes of potential effects

Longitudinal changes in outcome measures over 36 months are presented in Tables 2–3. For GDS and CBS, total scores, CBS sub scores and MCS raw, there were no differences between groups over 36 months. For PCS raw scores, the intervention group had higher scores at baseline compared to the control group, which, decreased over time. The difference between the groups at 24 months was 7.7 points (Confidence Interval [CI] = 1.19–14.2, *p* = 0.02), and at 36 months 6.29 points (CI =–0.27–12.8, *p* = 0.06), in favour of the control group. Regarding dimensions of the SF-36, there was a difference between the groups in favour of the control group, at 24 months for physical function (6.3 points, CI = 0.29–12.2, *p* = 0.04) and bodily pain (12.1 points, CI = 0.51– 23.7, *p* = 0.04), and at 36 months for general health perceptions (7.9 points, CI =–0.41–15.5, *p* = 0.03).

**Table 2:** Within- and between-group differences in depressive symptoms and caregiver burden among informal primary caregivers

Measure	Baseline value and within-group difference ^a^						Between-group difference ^a^	
	*n*	Control group, mean (SE)	*p*-value	*n*	Intervention group, mean (SE)	*p*-value	Mean (95% Confidence Interval)	*p*-value
**GDS (0–15)**								
*Baseline*	*31*	*3.2 (0.55)*		*35*	*2.2 (0.51)*			
5 mo	31	0.32 (0.53)	0.54	34	0.09 (0.50)	0.86	0.24 (–1.2–1.7)0	0.75
12 mo	29	0.61 (0.54)	0.26	35	0.37 (0.50)	0.46	0.24(–1.2–1.7)	0.75
24 mo	28	0.61 (0.55)	0.27	35	0.26 (0.50)	0.61	0.35 (–1.1–1.8)	0.64
36 mo	27	0.87 (0.57)	0.12	35	0.74 (0.50)	0.14	0.13 ( –1.3–1.6)	0.87
**CBS total score (0–4)**								
*Baseline*	*31*	*2.1 (0.10)*		*35*	*2.0 (0.09)*			
5 mo	31	0.05 (0.08)	0.57	33	0.09 (0.8)	0.26	–0.04 (–0.26–0.18)	0.72
12 mo	28	0.06 (0.08)	0.52	33	0.08 (0.8)	0.32	–0.02 (–0.25–0.20)	0.84
24 mo	24	0.10 (0.09)	0.28	31	0.003 (0.8)	0.97	0.09 (–0.14–0.33)	0.44
36 mo	19	0.15 (0.10)	0.11	24	0.18 (0.8)	0.04	–0.03 (–0.28–0.23)	0.83
**CBS General Strain (0–4)**								
*Baseline*	*31*	*2.3 (0.11)*		*34*	*2.2 (0.10)*			
5 mo	29	0.13 (0.11)	0.23	33	0.08 (0.10)	0.44	0.05 (–0.24–0.34)	0.74
12 mo	28	0.07 (0.11)	0.52	33	0.12 (0.10)	0.24	–0.05 (–0.34–0.24)	0.73
24 mo	24	0.15 (0.11)	0.20	31	-0.02 (0.10)	0.87	0.16 (–0.14–0.47)	0.29
36 mo	19	0.20 (0.12)	0.11	24	0.22 (0.11)	0.06	–0.02 (–0.34–0.31)	0.92
**CBS Isolation (0–4)**								
*Baseline*	*31*	*2.0 (0.13)*		*35*	*2.0 (0.12)*			
5 mo	31	0.11 (0.11)	0.33	33	0.04 (0.11)	0.72	0.07 (–0.24–0.37)	0.66
12 mo	28	0.13 (0.12)	0.27	33	0.06 (0.11)	0.58	0.07 (–0.24–0.38)	0.66
24 mo	24	0.15 (0.12)	0.23	31	0.11 (0.11)	0.30	0.03 (–0.29–0.35)	0.85
36 mo	19	0.19 (0.13)	0.15	24	0.30 (0.12)	0.01	–0.11 (–0.24–0.37)	0.66
**CBS Disappointment (0–4)**								
*Baseline*	*31*	*1.8 (0.11)*		*35*	*1.8 (0.10)*			
5 mo	31	0.03 (0.09)	0.78	32	0.12 (0.09)	0.16	–0.10 (–0.34–0.15)	0.44
12 mo	28	0.17 (0.09)	0.06	33	0.02 (0.09)	0.85	0.16 (–0.09–0.41)	0.22
24 mo	28	0.17 (0.10)	0.08	33	-0.03 (0.09)	0.73	0.20 (–0.06–0.47)	0.13
36 mo	19	0.24 (0.11)	0.03	24	0.17 (0.10)	0.09	0.07 (–0.21–0.36)	0.61
**CBS Emotional involvement (0–4)**								
*Baseline*	*31*	*2.2 (0.13)*		*35*	*2.0 (0.12)*			
5 mo	31	–0.09 (0.12)	0.46	33	0.02 (0.11)	0.86	–0.11 (–0.42–0.21)	0.51
12 mo	28	–0.15 (0.12)	0.23	33	-0.03 (0.11)	0.78	–0.11 (–0.43–0.21)	0.49
24 mo	24	–0.07 (0.13)	0.57	31	-0.08 (0.11)	0.49	0.01 (–0.33–0.34)	0.97
36 mo	19	–0.02 (0.14)	0.91	24	0.04 (0.12)	0.78	–0.05 (–0.41–0.31)	0.78
**CBS Environment (0–4)**								
*Baseline*	*31*	*1.9 (0.11)*		*35*	*1.7 (0.10)*			
5 mo	31	–0.07 (0.11)	0.56	33	0.18 (0.11)	0.09	–0.25 (–0.54–0.05)	0.11
12 mo	28	–0.06 (0.12)	0.62	33	0.20 (0.11)	0.06	–0.26 (–0.56–0.05)	0.10
24 mo	24	–0.06 (0.12)	0.61	31	0.09 (0.11)	0.42	–0.15 (–0.46–0.17)	0.36
36 mo	19	–0.01 (0.13)	0.94	24	0.14 (0.12)	0.25	–0.15 (–0.49–0.20)	0.41

GDS = Geriatric Depression Scale: higher scores indicate more depressive symptoms, CBS = Caregiver Burden Scale: higher scores indicate more burden

^a^ Performed using linear mixed models with least square means (follow-up–baseline values), adjusted for age and sex as fixed effects and the individual as a random effect

**Table 3:** Within- and between-group differences in health-rated quality of life among informal primary caregivers

Measure	Baseline value and within-group difference ^a^						Between-group difference ^a^	
	*n*	Control group, mean (SE)	*p*-value	*n*	Intervention group, mean (SE)	*p*-value	Mean (95% Confidence Interval)	*p*-value
**SF-36 MCSraw (0–100)**								
*Baseline*	*31*	*70.2 (3.4)*		*35*	*78.8 (3.2)*			
5 mo	31	0.37 (2.8)	0.90	35	–2.2 (2.7)	0.40	2.61 (–5.0–10.2)	0.50
12 mo	29	2.5 (2.9)	0.39	35	–2.1 (2.7)	0.42	4.64 (–3.1–12.4)	0.24
24 mo	28	0.89 (2.9)	0.76	35	–3.1 (2.7)	0.24	4.02 (–3.8–11.8)	0.31
36 mo	27	0.27 (3.0)	0.93	35	–5.3 (2.7)	0.045	5.62 (–2.2–13.4)	0.16
**SF-36 PCSraw (0–100)**								
*Baseline*	*31*	*69.2 (3.5)*		*35*	*80.5 (3.3)*			
5 mo	31	–1.2 (2.4)	0.61	35	–2.0 (2.2)	0.36	0.82 (–5.6–7.2)	0.80
12 mo	29	–0.78 (2.4)	0.75	35	–2.2 (2.2)	0.33	1.37 (–5.1–7.8)	0.67
24 mo	28	0.26 (2.5)	0.92	35	–7.4 (2.2)	0.001	7.70 (1.19–14.2)	0.02
36 mo	27	–1.3 (2.5)	0.60	35	–7.6 (2.2)	0.001	6.29 (–0.27–12.8)	0.06
**SF-36 Physical Function (PF) (0–100)**								
*Baseline*	*31*	*75.4 (3.7)*		*35*	*88.5 (3.5)*			
5 mo	31	–1.1 (2.1)	0.60	35	–3.0 (2.0)	0.14	1.8 (–4.0–7.7)	0.53
12 mo	29	–2.4 (2.2)	0.28	35	–2.7 (2.0)	0.18	0.29 (–5.6–66.3)	0.92
24 mo	28	0.05 (2.2)	0.98	35	–6.2 (2.0)	0.003	6.3 (0.29–12.2)	0.04
36 mo	27	–2.9 (2.3)	0.20	35	–6.0 (2.0)	0.004	3.1 (–3.0–9.1)	0.32
**SF-36 Role limitation due to physical health problems (RP) (0–100)**								
*Baseline*	*31*	*75.9 (4.4)*		*35*	*86.9 (4.1)*			
5 mo	31	–9.3 (4.0)	0.02	35	–2.1 (3.8)	0.57	–7.1 (–18.0–3.8)	0.20
12 mo	29	–8.2 (4.2)	0.047	35	–1.4 (3.8)	0.71	–6.8 (–17.9–4.2)	0.22
24 mo	28	–3.4 (4.2)	0.42	35	–8.8 (3.8)	0.02	5.4 (–5.8–16.5)	0.34
36 mo	27	–5.1 (4.2)	0.23	35	–11.8 (3.8)	0.002	6.7 (–4.5–17.9)	0.24
**SF-36 Bodily Pain (BP) (0–100)**								
*Baseline*	*31*	*62.8 (4.8)*		*35*	*74.9 (4.4)*			
5 mo	31	2.7 (4.2)	0.52	35	–0.80 (4.0)	0.84	3.5 (–7.9–14.9)	0.54
12 mo	29	4.7 (4.3)	0.27	35	–0.51 (4.0)	0.90	5.2 (–6.3–16.8)	0.37
24 mo	28	3.2 (4.4)	0.47	35	–9.0 (4.0)	0.02	12.1 (0.51–23.7)	0.04
36 mo	27	1.1 (4.4)	0.80	35	–6.2 (4.0)	0.12	7.3 (–4.4–19.0)	0.22
**SF-36 General Health perceptions (GH) (0–100)**								
*Baseline*	*31*	*62.7 (3.8)*		*35*	*72.5 (3.5)*			
5 mo	31	2.9 (2.7)	0.28	35	–2.2 (2.6)	0.39	5.0 (–2.3–12.4)	0.17
12 mo	29	2.8 (2.8)	0.30	35	–4.0 (2.6)	0.11	6.8 (–0.69–14.2)	0.07
24 mo	28	1.2 (2.8)	0.65	35	–5.8 (2.6)	0.02	7.1 (–0.43–14.5)	0.06
36 mo	27	1.6 (2.8)	0.56	35	–6.3 (2.6)	0.01	7.9 (–0.41–15.5)	0.03
**SF-36 Vitality (VIT) (0–-100)**								
*Baseline*	*31*	*54.2 (4.3)*		*35*	*65.3 (4.0)*			
5 mo	31	1.9 (3.3)	0.54	35	–4.2 (3.1)	0.16	6.2 (–2.8–15.2)	0.16
12 mo	29	0.26 (3.4)	0.94	35	–6.1 (3.1)	0.04	6.3 (–2.8–15.4)	0.15
24 mo	28	–1.2 (3.4)	0.70	35	–4.6 (3.1)	0.12	3.4 (–5.8–12.6)	0.44
36 mo	27	–0.27 (3.5)	0.94	35	–7.4 (3.1)	0.01	7.1 (–2.2–16.2)	0.11
**SF-36 Social Functioning (SF) (0–100)**								
*Baseline*	*31*	*78.6 (4.0)*		*35*	*85.5 (3.7)*			
5 mo	31	0.0 (3.9)	1.0	35	–3.2 (3.6)	0.37	3.2 (–7.3–13.7)	0.54
12 mo	29	1.9 (3.9)	0.62	35	–1.1 (3.6)	0.77	3.0 (–7.6–13.6)	0.57
24 mo	28	2.2 (4.0)	0.59	35	–4.3 (3.6)	0.24	6.4 (–4.2–17.1)	0.23
36 mo	27	2.4 (4.1)	0.55	35	–6.8 (3.6)	0.06	9.2 (–1.5–20.0)	0.09
**SF-36 Role limitations due to personal** **/emotional problems (RE) (0–100)**								
*Baseline*	*31*	*75.2 (4.6)*		*35*	*84.1 (4.3)*			
5 mo	31	–1.1 (4.7)	0.82	35	–0.95 (4.4)	0.83	–0.12 (–12.8–12.6)	0.99
12 mo	29	3.7 (4.8)	0.44	35	0.48 (4.4)	0.91	3.2 (–9.6–16.1)	0.62
24 mo	28	–0.02 (4.9)	1.0	35	–0.95 (4.4)	0.83	0.93 (–12.0–13.9)	0.89
36 mo	27	–3.0 (4.9)	0.54	35	–3.3 (4.4)	0.45	0.33 (–12.7–13.3)	0.96
**SF-36 Mental Health (MH) (0–100)**								
*Baseline*	*31*	*72.6 (3.1)*		*35*	*80.3 (2.9)*			
5 mo	31	0.61 (2.7)	0.82	35	–0.57 (2.6)	0.82	1.2 (–6.2–8.5)	0.75
12 mo	29	4.0 (2.8)	0.15	35	–1.9 (2.6)	0.47	5.9 (–1.5–13.3)	0.12
24 mo	28	2.7 (2.8)	0.34	35	–2.6 (2.6)	0.30	5.3 (–2.1–12.8)	0.16
36 mo	27	2.0 (2.8)	0.49	35	–3.9 (2.6)	0.13	5.9 (–1.7–13.4)	0.13

SF-36 = Short Form 36 –Health Survey, MCS raw= Mental Component Score raw (SF-36), PCS raw =Physical Component Score raw (SF-36). SF-36, all dimensions: higher scores indicate better status

^a^ Performed using linear mixed models with least square means (follow-up–baseline values), adjusted for age and sex as fixed effects and the individual as a random effect

## Discussion

This study, evaluating the feasibility of a rehabilitation program for people with dementia and their informal caregivers with focus on the caregivers, showed a high participation rate in follow-up assessments among caregivers over three years where missing assessments were mostly due to the person with dementia or caregiver being deceased. In the intervention group, the overall attendance of those choosing to participate in the group sessions was high. Furthermore, all caregivers were assessed and received interventions by the team staff during the study. The survey revealed that caregivers attending group sessions reported an improved ability to cope with the situation of being a caregiver and increased knowledge about supporting the person with dementia. In longitudinal analyses, the caregivers in both groups seemed to experience more depressive symptoms and higher burden over three years, and there were no differences between the intervention and control groups over 36 months. Regarding health-related quality of life, caregivers in the control group seemed stable while caregivers in the intervention group seemed to decline. There were differences between the groups in favour of the control group at 24 months in PCS, physical function, and bodily pain, and at the 36-month follow-up in general health perceptions.

Generally, caregivers fulfilled participation throughout the 36-month study period. Assessments and instruments seem feasible, as only one assessment was missing due to caregiver decline and there were few missing values that required imputation. Regarding participation in group sessions offered during the first 20 weeks, the participation rate was 66%, as not all caregivers chose to attend. A reason for this may be that the participants with dementia in the present study were a heterogenous group regarding dementia type and severity, and thus caregivers may have had varying needs at the time when group sessions took place. Although adult children were offered after-hours sessions, participation remained somewhat lower among adult children than that of partners (62% versus 71%). The caregivers who participated in group sessions had a high attendance rate, and in addition, medical records showed that supportive efforts were delivered to all caregivers during the intervention. Thus, although caregivers were also likely a heterogenous group, they all took part in supportive efforts.

There were no positive effects on the outcomes in favour of the intervention group, despite the intervention in the present study also containing rehabilitation efforts for the person with dementia. The persons with dementia in the intervention group received a substantial number of efforts compared to the control group [25], in addition to caregiver support. Regarding depressive symptoms and caregiver burden, there were only small non-significant differences between the groups, which do not seem clinically relevant [30, 48]. In analyses of the people with dementia in the MIDRED study, there were indications of clinical meaningful differences between the groups in gait, social [26] and physical activity, and neuropsychiatric symptoms, benefiting the intervention group [25]. These improvements in the person with dementia, in addition to the caregiver support, have the potential to impact burden placed on as well as the health of informal caregivers [9–12]. However, no such effect was seen in this pilot study. Regarding subscales of health-related quality of life, the control group generally increased or seemed stable, while the intervention showed a decline over time. The significant differences between the groups at the 24- and 36-month follow-up seem also clinically meaningful [49]. As dementia disorders are degenerative in nature, the observed differences in self-rated health are challenging to interpret or attribute to the intervention, given the time elapsed since the team-based efforts were implemented. Considering the results from the MIDRED-study, indicating people with dementia in the intervention group as continuing to be alive and living in the community longer than those in the control group, and being more physically [25] and socially active [26], informal caregivers may have faced increased demands and for a longer period. This interpretation is further supported by indications of higher levels of home-help services in the control group at 24 and 36 months (Additional file 3). Such increased demands may negatively influence caregivers’ health status and help explain some of the differences in caregivers’ health after two and three years. In the qualitative interviews after the 20-week intervention period, caregivers also describe that activities for the person with dementia could be experienced as leading to restrictions for the caregiver and their own needs being set aside [27]. In addition, the results may be influenced by regression towards the mean, especially for the subscales general health and bodily pain considering that the randomized groups developed in different directions over time, e.g. the intervention group with higher scores at baseline tended to decline over time.

The survey, which was completed while the rehabilitation was still ongoing, indicated that caregivers felt better equipped to support their relative with dementia. This is in line with previous studies indicating education and support to have the potential to positively influence caregivers [20–22]. In addition, the qualitative interviews, conducted during the last two weeks of the rehabilitation period in the MIDRED study revealed important positive experiences by those participating [23, 27]. The benefits reported in the survey as well as qualitative interviews could not be confirmed by the longitudinal data, and this could possibly be explained by factors related to study design. The intervention efforts in the present study were offered during prespecified time periods. The questionnaires about caregiver burden, depressive symptoms and self-rated health were filled out in between these periods, at a time when the dyad of caregiver and the person with dementia was referred to their usual care contacts, and staff team personnel returned to their regular positions. Because of this, participants in the present study may have experienced that support was withdrawn as the 20-week rehabilitation period ended. In the qualitative interviews, both caregivers and persons with dementia expressed worries about the future following the rehabilitation period, feeling unsure of how the future would turn out, and whether services from community and from healthcare would be delivered [23, 27]. In addition, caregivers described that the project filled a gap in the care that they and their relative with dementia had previously received from healthcare [27]. The experience that good formal care is lacking has been described earlier as leading to feelings of loneliness among caregivers [50]. Thus, it may be necessary for support to be offered over a longer period of time, with continuous contact based on individual needs.

The lack of clinical meaningful effects of the intervention in longitudinal assessments, which differed compared to positive indications in the survey and qualitative interviews, may also depend on the instruments used for evaluation. A discrepancy between methods has been described earlier in literature summarizing both quantitative and qualitative evaluations [51, 52]. Possible explanations have been proposed to be related to a lack of sensitivity of measurement instruments, inadequate target group orientation, short duration or dosage or interventions, or that observed social or relational benefits do not translate into measurable improvements [51, 52]. The present study included a heterogenous group of caregivers, both according to age but also with different relationships to the person with dementia, which led to a challenge in finding instruments suitable to all participants. The instrument used to evaluate depressive symptoms, for example, may be less sensitive among the younger caregivers since it is developed specifically for older adults [37]. Based on the positive indications from the survey and the qualitative studies, it would be relevant to also assess outcomes during ongoing intervention to gain a deeper understanding of effects of rehabilitation and caregiver support in areas regarding caregiver wellbeing, as well as sensitivity of instruments.

### Strengths and limitations

A strength of the present study is the documentation of the support provided to caregivers within the intervention group, which provides a description of the scope of the interventions. Since the study only had access to medical records of the person with dementia, we had limited knowledge regarding efforts given to participants in the control group, as well as the medical health situation for all caregivers. Greater insight into caregivers’ health could have enabled more person-centred support. Further, a limitation of the study was that the MIDRED study aimed for a fully powered RCT at study start, thus, no pre-specified feasibility criteria for pilot trials were established during the planning of the MIDRED study. In a complex intervention conducted in an underexplored area, it may be challenging to define a priori feasibility thresholds against which study feasibility can be judged. Assessing feasibility from a holistic perspective may be relevant in complex studies, where consideration is given to all collected data and to whether any modifications to the study protocol are needed. In the present study, an overall interpretation of the results was made, including recommendations for refining the assessments and intervention to support the implementation of a future definitive RCT. These recommendations were informed not only by the results of the present study but also by findings from the qualitative studies within the MIDRED study [23, 27].

## Conclusions

The multidimensional interdisciplinary rehabilitation program for community-dwelling people with dementia that incorporated education and support for informal caregivers, seems feasible in terms of caregivers’ participation in both the interventions and study evaluation over 36 months. Caregivers in the intervention group participating in education and group discussions, generally rated increased knowledge and coping strategies in the caregiver situation at completion. Over three years, caregiver burden and depressive symptoms seemed to increase in both groups. No indications of between-group differences were observed in caregiver burden and depressive symptoms, however several health-related quality of life subscales favoured the control group at 24 and 36 months. The results support further evaluation in an adequately powered randomized controlled trial. A future definite trial should refine the program further and offer continuous support over a longer period of time adapted to the individual. Assessments conducted during ongoing intervention period may offer greater insight into rehabilitation and caregiver-support effects, as well as instrument sensitivity. Lastly, the instrument for assessing depression should be appropriate for populations spanning a wide age range.

## Supplementary information

Below is the link to the electronic supplementary material.

Supplementary Material 1

Supplementary Material 2

Supplementary Material 3

## Data Availability

The datasets generated and/or analysed during the current study are not publicly available due to issues of individual privacy but are available from the corresponding author on reasonable request. Data can also be available from the Head of the Department of Community Medicine and Rehabilitation, Umeå University, for researchers who meet the criteria for access to confidential data. Contact: Department of Community Medicine and Rehabilitation, Umeå University, 901 87Umeå, Sweden. E-mail: studieadm.samrehab@umu.se.

## Abbreviations

ADL: activities of daily living
MIDRED: Multidimensional Interdisciplinary Rehabilitation in Dementia
RCT: randomized controlled trial
NPS: neuropsychiatric symptoms
CBS: Caregiver Burden Scale
SF: 36–Short Form 36–Health Survey
p: ADL–personal activities of daily living
i: ADL–instrumental activities of daily living
NPI: Neuropsychiatric Inventory
PCS: Physical Component Scale
MCS: Mental Component Scale
GDS: Geriatric Depression Scale
CI: Confidence Interval
