# Supplementary material for: Feasibility of a multidimensional interdisciplinary rehabilitation program for people with dementia and their informal primary caregivers – a randomized pilot trial with a caregiver focus
Source: BMC Geriatr. 2026 Oct 21;26:1326. doi: 10.1186/s12877-026-08532-1 (PMC13688808; doi:10.1186/s12877-026-08532-1)
Supplement: Supplementary file 1 [file 12877_2026_8532_MOESM1_ESM.docx]

**Table A2** Baseline characteristics of the persons with dementia in the MIDRED study (n =60)

| **Characteristic** | **Total n =60** | **Control group**  **n =29** | **Intervention group n =31** |
| --- | --- | --- | --- |
| ***Persons with dementia*** |  |  |  |
| Age, mean ± SD (range) | 79.0±6.4 (63–97) | 79.5±6.8 (66–97) | 78.5±6.0 (63–87) |
| Female sex, n (%) | 35 (58) | 15 (52) | 20 (65) |
| Dementia type, n (%) |  |  |  |
| - *Alzheimer´s disease* | 29 (48) | 13 (45) | 16 (52) |
| - *Vascular* | 11 (18) | 6 (21) | 5 (16) |
| - *Mixed Alzheimer’s disease and vascular* | 11 (18) | 6 (21) | 5 (16) |
| - *Other** | 9 (15) | 4 (14) | 5 (16) |
| Depression diagnosis, n (%) | 29 (48) | 13 (45) | 16 (52) |
| Antipsychotics, n (%) | 4 (7) | 0 (0) | 4 (13) |
| Antidepressants, n (%) | 19 (32) | 10 (35) | 9 (29) |
| No. prescribed drugs, mean ± SD (range) | 6.5±3.1 (1–15) | 6.6±3.1 (1–15) | 6.4±3.2 (1–14) |
| GDS-15 (0–15), mean ± SD (range) | 3.5±2.7 (0–11) | 3.17±2.4 (0–9) | 3.7±3.1 (0–11) |
| MMSE (0–30), mean ± SD (range) | 21.0±3.9 (9–30) | 21.1±3.6 (15–28) | 20.8±4.3 (9–30) |
| L&B P-ADL (6–30), mean ± SD (range) | 8.6±3.4 (6–21) | 8.8±4.0 (6–21) | 8.4±2.7 (6–17) |
| L&B I-ADL (8–31), mean ± SD (range) | 18.8±5.8 (8–28) | 18.7±6.3 (8–28) | 19.1±5.4 (10–28) |
| Independent in bladder management**, n (%) | 56 (93) | 26 (90) | 30 (97) |
| Independent in bowel management **, n (%) | 58 (97) | 28 (97) | 30 (97) |
| NPI (0–144), mean ± SD (range) | 9.7±10.4 (0–48) | 9.2±9.5 (0–48) | 10.2±11.3 (0–46) |
| NPI *cluster of symptoms,* mean ± SD (range) |  |  |  |
| - *Agitation/Hyperactivity (0-60)* | 3.1±4.9 (0–28) | 2.8±3.4 (0–14) | 3.4±5.9 (0–28) |
| - *Affective (0-*–*24)* | 2.3±3.7 (0–14) | 1.7±2.7 (0–11) | 2.8±4.7 (0–14) |
| - *Apathy (0*–*24)* | 2.3±3.7 (0–12) | 2.5±3.8 (0–12) | 2.1±3.6 (0–12) |
| - *Psychosis (0*–*36)* | 2.0±3.6 (0–15) | 2.2±3.9 (0–15) | 1.8±3.3 (0–13) |

* Other types of dementia include LBD, Parkinson´s dementia, combined vascular dementia, and LBD.
** Based on Functional Independence Measure score >5
*Note:* GDS = Geriatric Depression Scale, MMSE = Mini-Mental State Examination, L&B = Lawton and Brody scale, NPI = Neuropsychiatric Inventory, SD = Standard Deviation. For GDS and NPI, higher scores indicate more depressive symptoms and more neuropsychiatric symptoms, respectively. For MMSE and L&B, higher scores are better.
