# Supplementary material for: Feasibility of a multidimensional interdisciplinary rehabilitation program for people with dementia and their informal primary caregivers – a randomized pilot trial with a caregiver focus
Source: BMC Geriatr. 2026 Oct 21;26:1326. doi: 10.1186/s12877-026-08532-1 (PMC13688808; doi:10.1186/s12877-026-08532-1)
Supplement: Supplementary file 2 [file 12877_2026_8532_MOESM2_ESM.docx]

**Table A1** Content of the group sessions in the MIDRED study

| **Session no.** | **Title** | **Group leader(s) and participating professions** |
| --- | --- | --- |
| **1** | *Information and presentation of content and aim of group sessions* | Social worker, nurse |
| **2** | *The caregiver situation*  *Social services*  *Support in the community*  *Economy* | Social worker, nurse |
| **3** | *Dementia disorders*  *Pharmaceutical treatment*  *Neuropsychological symptoms in dementia* | Nurse, specialized physician |
| **4** | *Psychological reactions in dementia disorders*  *Behavioural response* | Nurse, neuropsychologist |
| **5** | *Physical impairments*  *Physical activity and guidelines*  *Activities of daily life* | Social worker, physiotherapist, occupational therapist |
| **6** | *Nutrition and oral health*  *Evaluation of the sessions* | Social worker, nurse, dietician, dental hygienist |
