# Supplementary material for: Feasibility of a multidimensional interdisciplinary rehabilitation program for people with dementia and their informal primary caregivers – a randomized pilot trial with a caregiver focus
Source: BMC Geriatr. 2026 Oct 21;26:1326. doi: 10.1186/s12877-026-08532-1 (PMC13688808; doi:10.1186/s12877-026-08532-1)
Supplement: Supplementary file 3 [file 12877_2026_8532_MOESM3_ESM.docx]

**Table A3** Social services according to decisions by the municipality for people with dementia in the MIDRED study,
presented for control and intervention group

|  | **Group** | **Baseline**  C: n =29,  Int: n =31 | **5 months**  C: n =28,  Int: n =28 | **12 months**  C: n =25,  Int: n =27 | **24 months**  C: n =22 ,  Int: n =20 | **36 months**  C: n =15,  Int: n =17 |
| --- | --- | --- | --- | --- | --- | --- |
| **Home-help services,** n (%) | Control | 9 (31) | 9 (32) | 11 (44) | 10 (46) | 7 (47) |
|  | Intervention | 11 (36) | 10 (36) | 11 (41) | 7 (35) | 4 (24) |
| **Home-help services,** h/w, mean ± SD (range) | Control | 2.22 5.56 | 2.62±6.06 | 2.85±5.51 | 3.49±5.18 | 3.40±7.69 |
|  | Intervention | 0.75±1.42 | 1.27±2.51 | 1.98±2.86 | 2.49±4.67 | 1.26±3.02 |
| **Honorary for caregiver, p-ADL,** n (%) | Control | 1 (3) | 0 | 0 | 0 | 0 |
|  | Intervention | 3 (9) | 3 (9) | 4 (13) | 3 (12) | 5 (25) |
| **Honorary for caregiver, p-ADL,** h/w, mean ± SD (range) | Control | 0.18±0.97 | 0 | 0 | 0.64±3.00 | 0 |
|  | Intervention | 0.58±2.30 | 1.0±3.9 | 1.94±5.21 | 2.04±6.40 | 3.28±7.50 |
| **Day-centre activities, n (%)** | Control | 4 (14) | 4 (14) | 4 (16) | 5 (23) | 4 (27) |
|  | Intervention | 5 (16) | 10 (36) | 10 (37) | 10 (50) | 7 (42) |
| **Alarm system, n (%)** | Control | 6 (21) | 5 (18) | 5 (20) | 6 (27) | 3 (20) |
|  | Intervention | 9 (29) | 9 (32) | 8 (30) | 4 (20) | 2 (12) |
| **Food delivery, n (%)** | Control | 3 (10) | 3 (11) | 1 (4) | 1 (5) | 1 (7) |
|  | Intervention | 3 (10) | 4 (14) | 3 (11) | 2 (10) | 1 (6) |
| **Accompanying services, n (%)** | Control | 1 (3) | 1 (4) | 1 (4) | 0 | 0 |
|  | Intervention | 4 (13) | 4 (13) | 3 (11) | 2 (10) | 2 (12) |
| **Assistance in self-care, n (%)** | Control | 0 | 1 (4) | 2 (8) | 2 (9) | 2 (13) |
|  | Intervention | 1 (3) | 1 (4) | 5 (19) | 2 (10) | 0 |

*Note:* C= control group, Int = intervention group, p-ADL = personal activities of daily living. Home-help services summarize efforts from
social services in ordinary housing and include assistance in p-ADL, household chores, meal support, purchases, cleaning, and laundry.
